# Effectiveness and Acceptability of Telerehabilitation in Physical Therapy during COVID-19 in Children: Findings of a Systematic Review

**DOI:** 10.3390/children8121101

**Published:** 2021-11-29

**Authors:** Asma Alonazi

**Affiliations:** Department of Physical Therapy and Health Rehabilitation, College of Applied Medical Sciences, Majmaah University, Riyadh 11952, Saudi Arabia; a.alonazi@mu.edu.sa

**Keywords:** telerehabilitation, physical therapy, children, pediatric, COVID-19, systematic review

## Abstract

The goal of this systematic review was to determine the efficacy and acceptability of telerehabilitation in physical therapy (PT) and parental acceptance during the COVID-19 pandemic in children. In 2021, an electronic search of academic articles was performed using databases such as Embase, PubMed and Scopus. One-hundred and one articles did not satisfy the eligibility criteria after deleting duplicates and reviewing abstracts, while 16 papers did not meet eligibility after reviewing complete texts. Hence, full texts for 13 articles were retrieved, which were incorporated in the review. All these studies were observational studies assessing the effectiveness and acceptability of telerehabilitation in PT required for diverse conditions in children during the COVID-19 pandemic. All included studies revealed a positive effect of telerehabilitation in PT during the COVID-19 pandemic in children with different conditions. Moreover, the included studies revealed that both rehabilitation professionals and parents or caregivers of children were satisfied with the telerehabilitation services provided remotely. Thus, telerehabilitation appears to be a suitable and convenient strategy to offer remote services to children in need but cannot visit in person due to COVID-19. The existing evidence shows that telerehabilitation can be considered effective for children who need PT for any health condition mainly during the pandemic. However, due to the dearth of studies in this area, exploring this topic is recommended mostly in low-middle-income countries with poor access to health care services and limited resources.

## 1. Introduction

Rehabilitation is vital for enhancing people’s capacity to live and perform necessary and daily routine activities or work and to improve the quality of life [1,2]. Professionals working with the rehabilitation of children with different disabilities need to collaborate with families and caregivers to share information and develop the capacity required to take care of their children [3,4]. Simultaneously, available technologies offer a variety of options for children’s care, paticularly for those who lack access to care during a crisis, such as the COVID-19 pandemic [5,6]. Recently, with the help of telecommunication, networks can be used to provide required health services remotely without the physical presence of health care professionals [7]. These technologies can improve access to required health care services for children in their homes in a cost-effective way [8,9]. Such technologies can be utilized in diverse methods in improving children’s health, mainly those who require physical therapy (PT) [10,11]. Telemedicine or telehealth or digital health are different terms used to provide care to patients at a distance [12,13]. Telerehabilitation, for example, is a term often used in the literature to describe rehabilitation experts who provide health treatments remotely via telecommunication technology [14,15]. Such services could either consist of using some simple strategies such as contacting parents for their children via phone or emails or complex techniques such as installing intricate equipment at home and guiding parents to use the same equipment at home [14,15].

In addition, during the COVID-19 crisis, the World Confederation for Physical Therapy (WCPT) and the International Network of Physiotherapy Regulatory Authorities (INPTRA) produced a report in 2020 on digital PT practice and offered several recommendations about PT [16,17]. First, according to the report, physical therapists need to consider children’s health conditions and assess whether that condition can be addressed by digital therapy. The report also emphasizes the importance of PT and the risks against the benefits of in-person appointments due to distance and cost. Finally, when deciding between in-person and virtual visits, consider the presence of caregivers and their availability to help the child, according to [16,17].

Despite the availability of telehealth and digital health and its vital role in improving children’s health, mainly during the COVID-19 crisis, rehabilitation professionals have relatively been in the infancy stage in embracing telerehabilitation services [18,19]. Few systematic reviews and meta-analyses have demonstrated the effectiveness and efficacy of telerehabilitation following some upper and lower limb interventions or for patients suffering from stroke with promising findings [20]. However, because the majority of these reviews were conducted on older populations or before the COVID-19 crisis, it is uncertain whether telerehabilitation in PT is effective for children during the COVID-19 crisis [20].

Furthermore, telerehabilitation and its importance are highlighted in the literature; however, none of the reviews or meta-analyses focus on synthesizing and reviewing studies conducted on the pediatric population mainly during the COVID-19 crisis [21,22]. Thus, the significance of telerehabilitation in PT, as well as its effectiveness in children and parental acceptability, must be further investigated, particularly during the global pandemic [23,24]. Synthesizing evidence from available literature on the efficacy or effectiveness of telerehabilitation services for children would provide avenues to use such services cost-effectively. This review would be unique from previously conducted reviews that have focused on adult patients or patients with specific health conditions such as stroke or breast cancer [25,26]. Telerehabilitation and the satisfaction of children and their parents are the two key variables considered in this review. Any study that has examined an intervention incorporates telerehabilitation as a core component, and the intervention that has been delivered remotely is considered telerehabilitation. Given the dearth of evidence on the effectiveness of telerehabilitation in PT during COVID-19 in children and interdisciplinary elements associated with the needs of children, this systematic review was undertaken to assess the effectiveness and acceptability of telerehabilitation in PT and its acceptance by parents during the COVID-19 crisis in children.

## 2. Materials and Methods

This review was meant to rigorously appraise, synthesize and aggregate current research on the effectiveness of telerehabilitation in PT and parental acceptance during the COVID-19 crisis. This review was conducted using a new PRISMA (Preferred Reporting Items for Systematic Reviews and Meta-Analyses) checklist that provides guidelines for reporting the systematic review, and the details of all items are provided in Table 1 [27].

### 2.1. Inclusion and Exclusion Criteria

An electronic search was performed systematically on the effectiveness of telerehabilitation in PT and its acceptance by parents during the COVID-19 crisis in children both in high and low-middle income countries. The World Bank’s 2018 country classification was used to define a high and low-middle-income country [28]. To answer the research question, a study was considered eligible if it considered telerehabilitation in PT for any medical or surgical condition among children aged less than 18 years, and it had to be an original research study published in English during the COVID-19 era in both developed and developing countries. On the contrary, if any study that had explored the effectiveness of telerehabilitation in PT and its acceptance by parents among adults or before the concept of COVID-19 or that are not published in the English language were excluded from the review. Moreover, articles that did not focus on PT were excluded. In addition, letters to the editor, grey literature secondary data and case reports were excluded.

### 2.2. Information Sources and Search Strategy

In 2021, a search of publications published recently during the COVID-19 era was completed. Electronic databases including Embase, PubMed and Scopus were searched. An independent search was carried out to screen and review the results of relevant studies. The primary exposure of interest was telerehabilitation in PT during COVID-19 and that therapy could be for any child’s health condition. Relevant Medical Subject Heading (MeSH) key terms were used to access the relevant articles. The most common key search terms included “telerehabilitation AND children,” “telerehabilitation in physical therapy,” “telerehabilitation in physical therapy AND children,” “telerehabilitation in physical therapy AND COVID-19 in the pediatric population,” “telerehabilitation in physical therapy AND global pandemic of COVID-19 AND kids,” “telerehabilitation in physical therapy AND global pandemic AND children,” “developing countries,” and developed countries. In addition, main concepts such as telerehabilitation in physical therapy, COVID-19 vs. global pandemic, or pandemic crisis were used to obtain pertinent research papers. Then, these major concepts were combined by using combinations (AND; OR) relevant to the research question. An example of a complete search strategy included the following: “telerehabilitation in physical therapy AND global pandemic AND children OR kids.” Furthermore, in order to identify more relevant articles, truncation (*) with a similar root word was employed. Search restrictions with filters were employed on the language (English), publication period and age category to include eligible studies in the search.

### 2.3. Data Abstraction and Quality Assessment

The appropriate research studies were imported into an EndnoteTM file (reference manager software), where each study was reviewed individually and duplicates were identified and removed using the same program. The abstracts that did not explicitly explore the study objective were not reviewed, and this was followed by retrieving and reviewing the full-text articles of the appropriate. A standardized proforma was used to abstract and summarize the articles that met the required eligibility criteria. In addition, the references of all relevant studies were also assessed to evade missing any useful studies.

The author’s reference, publication year, title, total sample size, sample size by gender if relevant, kind of intervention and age group were all included in the abstracted data. For all cross-sectional studies, distinct Newcastle–Ottawa Scales were used to assess the quality and risk of bias of each eligible full-text publication [29]. According to the Newcastle–Ottawa Scale for cross-sectional studies, three main domains were to be assessed, including selection, comparability and outcome ascertainment. The maximum score for selection was 5, which was based on the representativeness of the study sample, sample size and its justification, response rate and ascertainment of the exposure. Likewise, the maximum score for comparability was two, based on the adjustment of potential confounders and additional factors in advanced analysis. Lastly, the maximum score for the outcome was three based on the assessment method for the outcome and a statistical test to analyze the results. The total score based on all these domains was ten, and each eligible study was assigned a score representing the quality of that study. Good studies were given scores of between 7and 10 points, satisfactory studies had scores of between 5 and 6 points, and those that scored were from 0 to 4 points were considered unsatisfactory.

## 3. Results

### 3.1. Findings of the Search Strategy

**Figure 1:**
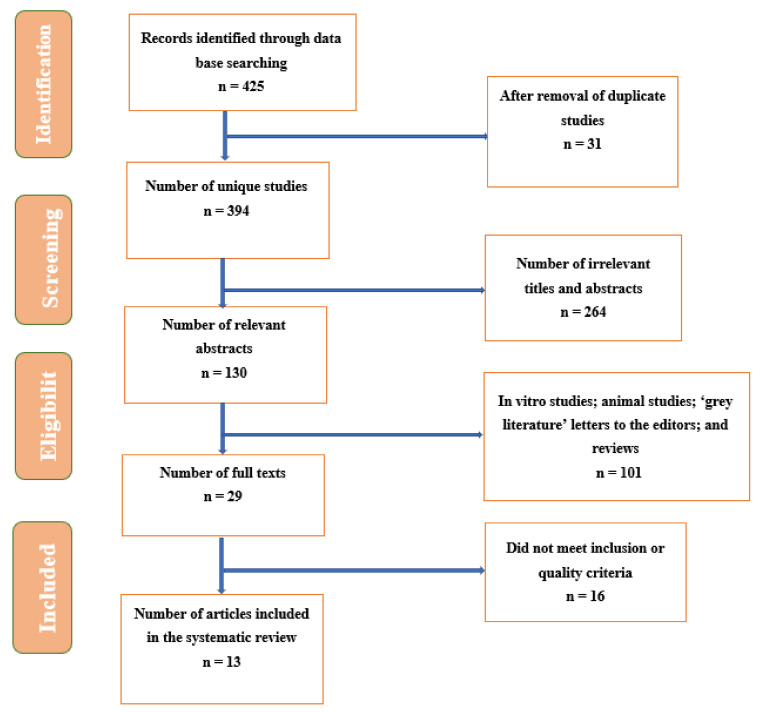
Flowchart summarizing the identification and selection of papers for systematic review.

The selected publications were initially screened by titles, then by abstracts, and finally by full-text articles. As a result, the first search identified 425 citations in the identified databases, with 31 duplicates that were removed. Of the remaining 394 unique studies, 130 relevant abstracts were reviewed. While reviewing the abstracts, 101 articles did not meet the eligibility criteria, and 16 did not meet eligibility after reviewing full texts. Hence, full texts for 13 articles were retrieved, which were incorporated in the review, as shown in Figure 1.

### 3.2. Characteristics of the Eligible Studies

With the exception of one observational retrospective study, all of these 13 investigations were conducted as surveys or cross-sectional studies. The sample size of all included research studies varied between 36 and 514. Some of the studies (*n* = 2) included both genders, while four studies did not report the gender of the study’s participants. The age of study participants was between 1 to a maximum of 18 (Table 2). Since it was an era of COVID-19, almost all studies were performed in 2021 except one study undertaken in 2020. Almost 50% of the included studies were performed in Italy, 20% were in Poland, and 15% in were Canada and the USA. This indicates that 100% of the studies were from developed countries.

The included studies provided telerehabilitation to children for various conditions, including autism spectrum disorders; cerebral palsy; Rett genetic syndrome; neuromuscular diseases; three with rare genetic or malformation syndromes; extremely premature musculoskeletal and neurodevelopment problems; and psychomotor or cognitive delay conditions. Almost all studies passed the quality criterion for risk of bias evaluation. Most studies, on the other hand, did not disclose the sampling technique or justify the sample size, and others did not account for crucial confounders.

### 3.3. Synthesis and Review of Findings Summarizing the Effectiveness and Acceptability of Telerehabilitation in PT during COVID-19 in Children

Overall, all included studies revealed the positive effect of telerehabilitation in PT during COVID-19 in children with different conditions. Moreover, the included studies revealed that both rehabilitation professionals and parents or caregivers of children were satisfied with the telerehabilitation services provided remotely. For instance, a study conducted by Sobierajska-Rek et al., 2021, on 152 children who have Duchenne muscular dystrophy found an average rating for satisfaction with the therapy of 4.7 out of 5 and 4.78 out of 5 for intelligibility [30]. In addition, around 83% of the study participants reported performing exercises, and their caregivers mentioned that it was feasible for their children to perform the recommended exercises a few times a week or daily [30].

Likewise, Pamela Frigerio, 2021, surveyed 128 families with children aged 0 to 14 suffering from neurodevelopmental disabilities [31]. The authors found that almost 80.5% of the caregivers showed their satisfaction with telerehabilitation [31]. The authors also found that more than 50% of the families mentioned a higher satisfaction score with telerehabilitation [31]. Similarly, another study was undertaken by A. Sobierajska-Rek et al. in 2021 on 69 children with Duchenne muscular dystrophy [32]. The authors concluded that physiotherapy could be continued as home-based rehabilitation because the parents or the caregivers accepted the instructions or videos provided remotely to a greater extent [32].

Tanner, Grinde and McCormick, 2021 conducted a cross-sectional study in Canada on 86 children with different conditions who required PT. The authors included both boys and girls in their study sample [33]. The study results illustrated that the Canadian Occupational Performance Measure (COPM) is considered a feasible measure that is perceived positively by professional pediatric therapists [33]. Around 83% of the therapists agreed or strongly agreed that COPM is easy to use and reasonable in a given period of time and can be used with children of different conditions, as shown in Table 3 [33].

A retrospective observational study was performed in the USA by Bican et al., 2021, on 514 children with a mean age of 4.3 ± 3.5 years. The researchers used physical or occupational therapy with 938 video visits and 150 telephone encounters for children with different conditions such as Musculoskeletal impairment, cerebral palsy, movement disorder, delayed milestones, feeding difficulties and prematurity or low birth weight. The study findings demonstrated that telerehabilitation services were received by 83.4% of the children, and most occupational and physical therapists (69.1%) agreed that telerehabilitation is as effective as in-person care. Around 93% of the therapists reported that caregivers were available during the sessions and were actively participating [34]. Likewise, Romano, Di Rosa, Tisano, Fabio and Lotan, 2021 studied children with Rett syndrome in Italy [35]. The study sample only included girls, and the rehabilitation program consisted of 47 goals with a customized program for each participant. The results showed that out of the total 47 rehabilitation goals set, 78.7% were achieved, with around 76.9% of the children’s gross motor function improved with a modest effect (0.604) [35]. Parents and caregivers rated the telerehabilitation program satisfactorily with a score of 4.4/5, and general satisfaction was 4.5/5, with adherence to the program being 4.4/5 [35] (Table 3).

Similarly, Provenzi et al., 2021, undertook a cross-sectional survey of 36 children in Italy, including 25 boys and 11 girls [37]. Researchers tested the online Rehabilitation of Children during the Epidemic (EnFORCE) telehealth program that included case-specific, tailored telehealth sessions and parental support and child rehabilitation sessions. The findings showed that >80% of the parents mentioned that their children benefited from the program during lockdown [37]. Moreover, around 86 to 95% of the parents reported increased feelings of engagement, perceived support and self-relevance [37]. In Italy, one study by Iannizzotto, Nucita, Fabio, Caprì and Lo Bello, 2020, was focused on 300 children with Rett genetic syndrome with a mean age of 11.31 ± 4.8 years [42]. The study’s preliminary results reveal that videoconferencing software is promising and can be used on a larger base cost effectively without relying on expensive and complicated devices where children can remotely communicate.

A transversal observational study conducted by Assenza et al., 2021, in Italy on 138 children with different musculoskeletal and neurodevelopmental problems tested the effects of physical, speech, occupational, and cognitive-behavioral therapy using telerehabilitation [40]. The findings revealed a correlation among caretakers of kids aged 0–3 with feeling overwhelmed with distance care (OR = 3.27), low perception of telerehabilitation for improving objectives (OR = 6.51), and a great perception of feeling supported in establishing regular activity (OR = 2.96) [40]. It was concluded that telerehabilitation could be a helpful strategy during a global pandemic [40]. A retrospective chart review and survey was conducted by K. Tanner et al. in 2020 on 767 children with different conditions who required physical, occupational and speech therapy [41]. The researchers used telerehabilitation services (speech-language pathology, developmental occupational and physical therapies and sports and orthopedic therapies). Study findings revealed a high satisfaction was found with 98.97% of the positive responses. Additionally, 73.5% of the pre-pandemic patients were returned after implementing telerehabilitation services [41].

Krasovsky et al., 2021, conducted a study in Israel, having focus group discussions with all experts from the field [36]. Overall, the results are in favor of transitioning to telerehabilitation for the pediatric population. Three components, including a child, parent and sessions, were found to explain 71.3% of the variance in The Clinician Evaluation of Telerehabilitation Service. Therapists stated that their capacity to establish therapeutic relationships outweighed their ability to achieve other objectives. According to the families, the therapist was highly involved in providing therapy to children regardless of the type of treatment [36].

Similarly, Hall et al. conducted a study in the USA to study the facilitators and barriers influencing the effectiveness of telerehabilitation during COVID-19 [38]. The study found that higher degrees of engagement and access to telehealth with stable technology are considered crucial factors for the effectiveness of telehealth. This telehealth model is supported and correlates with internet availability and good connection and the interaction between child and caregiver, and family resilience may play a vital role in moving towards telerehabilitation during the COVID-19 crisis [38]. Gagnon et al. performed a study in Canada to evaluate the feasibility of delivering a home exercise-based programs to children with Arthrogryposis multiplex congenital [39]. The study found that participants performed a home exercise program almost twice a week (Mean: 2.04 with 95% CI of 1.25 to 4.08), and they were satisfied with the approach. Out of 15 goals that were set at the beginning of the program, 12 goals were achieved. The study found a statistically significant improvement in the pain and comfort for pediatric outcomes (*p*-value: 0.048) on the physical activity questionnaire [39].

## 4. Discussion

This systematic review was undertaken by reviewing and synthesizing the evidence available on the effectiveness of telerehabilitation in PT during COVID-19 in children. Generally, the findings based on the included studies revealed the positive effect of telerehabilitation in PT during COVID-19 in children with different conditions. Moreover, included studies revealed that both rehabilitation professionals and parents or caregivers of children were satisfied with the telerehabilitation services provided remotely. In addition, the included studies provided telerehabilitation to children suffering from different conditions such as autism spectrum disorders; cerebral palsy; Rett genetic syndrome; neuromuscular diseases; three with rare genetic or malformation syndromes; extremely premature musculoskeletal and neurodevelopment problems; and psychomotor or cognitive delay conditions.

These findings are consistent with the effectiveness of telerehabilitation in PT among adults [43,44]. For instance, findings from a systematic review have demonstrated that telerehabilitation-based consultation for musculoskeletal pain is possible with respect to concurrent validity and interrater and interrater reliability for assessing peripheral joints and the spine, with psychometric properties ranging between good and excellent for various clinical conditions such as pain, swelling on the body, muscular strength, body’s balance, gait and range of motion (active and passive) [43,45]. Consistent findings from these systematic reviews and the current systematic review reveal that telerehabilitation has several advantages for rehabilitation professionals as it allows them to continue providing care for various conditions without any interference. They can counsel and educate patients remotely with reasonable consistency in a more comfortable environment at their homes, complete an evaluation and assess patients, plan a tailored and customized therapeutic exercise intervention and assess patients’ progress by delivering them constant advice and feedback under supervision [46,47]. These benefits of telerehabilitation are both for children as well as their physical therapists, as it can reduce the number of readmissions and hospitalizations, improve access to services cost-effectively and it is timesaving [46,47]. Furthermore, previous systematic reviews and meta-analyses conducted on adults or patients with stroke or breast cancer patients reveal findings analogous to the current findings, which reinforces the utility of telerehabilitation services for all ages and all genders who need those services [22,25,26].

These findings are confirmed by a recently conducted rapid but comprehensive review that attempted to synthesize the literature on the effectiveness of telerehabilitation, including different clinical aspects and conditions in the responsibility of physical therapists. The findings of this rapid review may be widely relevant and applicable today when we face the COVID-19 pandemic crisis [1]. These findings provide us with confidence that effective telerehabilitation services exist that can be safely used in the event of a crisis such as the COVID-19 pandemic. Furthermore, the horizon of these services can be broadened as these are not restricted to one condition such as musculoskeletal disorders or stroke but also can be conducted for cardiovascular rehabilitation, neurorehabilitation and respiratory rehabilitation, for example. This also suggests that consistency in providing treatment should be there, even in a crisis [1].

Despite the increased interest in telerehabilitation in general and specifically for the pediatric population, current guidelines on safety, conditions, standards and practice requirements in providing telerehabilitation services must be followed. In this regard, the existing literature focuses on clinical and technical principles while simultaneously highlighting the ethical aspects of services provided. More precisely, existing evidence emphasizes that organizations or professionals involved in providing such services should be licensed, trained and certified in order to provide such services [48]. Furthermore, such guidelines place a strong emphasis on the privacy and confidentiality of children who are suffering from various conditions that necessitate PT. Furthermore, professionals providing telerehabilitation should receive continuing education to upgrade their skills and know what interventions to provide to children as per their health conditions. An important point to consider is that each intervention may not suit all children. Hence, interventions need to be tailored according to the needs of children [48]. Finally, with respect to ethics, organizations and professionals providing telerehabilitation services to children should comply with the professional code of ethics, and there should not be any conflict of interests with the service provision [48].

### 4.1. Strengths and Limitations

To our knowledge, this is the first study of its sort focusing on the population of children that requires PT using virtual or remote services during COVID-19. Therefore, the findings of this review can help physicians, pediatricians and physical therapists to make informed decisions about providing services to children during a time of crisis. Furthermore, an updated PRISMA checklist was used to undertake this review and assessed the quality of the studies. The findings of this review can provide a framework for clinicians, physiotherapists and policymakers in providing continuous care to children even during a pandemic. Despite these strengths, the findings need to be interpreted with caution due to some caveats associated with the individual studies. First, the findings of the present review suggest that most of the studies on effectiveness and acceptability are from developed countries. Therefore, one needs to be cautious while extrapolating these results to other settings with limited resources and poor access to care and sophisticated technologies. Additionally, findings need to be interpreted cautiously based on these studies because not all the studies were appropriately internally valid, and most of these did not adjust for potential confounders.

Almost all the included studies were cross-sectional study designs. These study designs do not help establish temporality between risk factors and outcomes. Second, most of the studies did not select study participants randomly, which may bias their study findings. Third, due to the observational nature of study designs, the issue of unmeasured confounding can always persist. However, one may overcome this issue by having an explicit theory about the potential confounders and identifying confounders using causal diagrams such as direct acyclic graphs. These graphs can help a researcher identify a minimum set of variables that need to be adjusted as potential confounders and may be highly correlated with unmeasured confounders. In this manner, the problem of unmeasured confounders can be addressed to a greater extent in observational studies. While the overall quality of studies was satisfactory, not all studies had a lower risk of bias, and there were some epidemiological issues with individual studies that should not be ignored. Furthermore, the included studies in the review were observational, and there is a dearth of randomized controlled trials in this area, which are considered gold standard study designs in assessing the efficacy and effectiveness of any intervention on the outcome of interest.

### 4.2. Future Implications for Clinical Practice and Research

Despite the above-mentioned caveats, the existing evidence suggests that telerehabilitation services can be used for children who need PT remotely mainly during the pandemic. Such an innovative approach is crucial mainly during the pandemic era (COVID-19), where the frequency of in-person rehabilitation services has plummeted. Regardless of inconclusive or low-quality evidence, clinicians should not ignore the utility of telerehabilitation, especially in settings where in-person or center-based rehabilitation is a challenge. Telerehabilitation appears to be a suitable and convenient strategy for offering remote services to children in need but cannot visit in person due to the COVID-19 crisis. Due to the dearth of evidence on this topic, clinical randomized controlled trials are recommended in the future to explore the effectiveness of telerehabilitation. Since our focus was during COVID-19, not many studies have been conducted or published in this area so far. Therefore, the exploration of this topic is recommended for low-middle-income countries with poor access to health care services and limited resources.

## 5. Conclusions

Both rehabilitation specialists and parents or carers of children appear to be satisfied with the telerehabilitation services supplied to their children remotely, according to the findings of the review. Furthermore, the findings highlighted that telerehabilitation interventions must be tailored to the specific needs of children, as one size does not fit all, and this may be especially relevant during the COVID-19 epidemic or other emergencies when children have restricted access to PT services. The included studies provided telerehabilitation to children suffering from different conditions such as autism spectrum disorders; cerebral palsy; Rett genetic syndrome; neuromuscular diseases; three with rare genetic or malformation syndromes; extremely premature musculoskeletal and neurodevelopment problems; and psychomotor or cognitive delay conditions.

## Figures and Tables

**Table 1:** PRISMA checklist used to undertake the review.

Section and Topic	Item	Checklist Item	Location Where Item Is Reported
TITLE			
Title	1	Identify the report as a systematic review.	Page 1
ABSTRACT			
Abstract	2	See PRISMA 2020 for abstracts checklist.	Page 1
INTRODUCTION			
Rationale	3	Describe the rationale for the review in the context of existing knowledge.	Page 2
Objectives	4	Provide an explicit statement of the objective(s) or question(s) the review addresses.	Page 2 and 3
METHODS			
Eligibility criteria	5	Specify the inclusion and exclusion criteria for the review and how studies were grouped for the syntheses.	Page 3
Information sources	6	Specify all databases, registers, websites, organisations, reference lists and other sources searched or consulted to identify studies. Specify the date when each source was last searched or consulted.	Page 3 and 4
Search strategy	7	Present the full search strategies for all databases, registers and websites, including any filters and limits used.	Page 3 and 4
Selection process	8	Specify the methods used to decide whether a study met the inclusion criteria of the review, including how many reviewers screened each record and each report retrieved, whether they worked independently, and details of automation tools used in the process if applicable.	Page 4
Data collection process	9	Specify the methods used to collect data from reports, including how many reviewers collected data from each report, whether they worked independently, any processes for obtaining or confirming data from study investigators, and details of automation tools used in the process if applicable.	Page 4
Data items	10a	List and define all outcomes for which data were sought. Specify whether all results that were compatible with each outcome domain in each study were sought (e.g., for all measures, time points and analyses), and if not the methods used to decide which results to collect should be specified.	Page 4 and 5
	10b	List and define all other variables for which data were sought (e.g., participant and intervention characteristics and funding sources). Describe any assumptions made about any missing or unclear information.	Page 4 and 5
Study risk of bias assessment	11	Specify the methods used to assess risk of bias in the included studies, including details of the tool(s) used, how many reviewers assessed each study and whether they worked independently, and if applicable details of automation tools used in the process.	Page 4
Effect measures	12	Specify for each outcome the effect measure(s) (e.g., risk ratio and mean difference) used in the synthesis or presentation of results.	NA
Synthesis methods	13a	Describe the processes used to decide which studies were eligible for each synthesis (e.g., tabulating the study intervention characteristics and comparing against the planned groups for each synthesis (item #5)).	NA
	13b	Describe any methods required to prepare the data for presentation or synthesis, such as handling of missing summary statistics or data conversions.	NA
	13c	Describe any methods used to tabulate or visually display results of individual studies and syntheses.	NA
	13d	Describe any methods used to synthesize results and provide a rationale for the choice(s). If meta-analysis was performed, describe the model(s) and method(s) that identify the presence and extent of statistical heterogeneity and software package(s) used.	NA
	13e	Describe any methods used to explore possible causes of heterogeneity among study results (e.g., subgroup analysis and meta-regression).	NA
	13f	Describe any sensitivity analyses conducted to assess robustness of the synthesized results.	NA
Reporting bias assessment	14	Describe any methods used to assess risk of bias due to missing results in a synthesis (arising from reporting biases).	NA
Certainty assessment	15	Describe any methods used to assess certainty (or confidence) in the body of evidence for an outcome.	NA
RESULTS			
Study selection	16a	Describe the results of the search and selection process, from the number of records identified in the search to the number of studies included in the review, ideally using a flow diagram.	Page 4 and Figure 1
	16b	Cite studies that might appear to meet the inclusion criteria but that were excluded and explain why they were excluded.	Page 5 and Figure 1
Study characteristics	17	Cite each included study and present its characteristics.	Page 5 and 8
Risk of bias in studies	18	Present assessments of risk of bias for each included study.	Page 8
Results of individual studies	19	For all outcomes, present, for each study: (a) summary statistics for each group (where appropriate) and (b) effect estimates and its precision (e.g., confidence/credible interval), ideally using structured tables or plots.	Page 8 to 13
Results of syntheses	20a	For each synthesis, briefly summarise the characteristics and risk of bias among contributing studies.	Page 8 to 13
	20b	Present results of all statistical syntheses conducted. If meta-analysis was conducted, present the summary estimate for each and its precision (e.g., confidence/credible interval) and measures of statistical heterogeneity. If comparing groups, describe the direction of the effect.	NA
	20c	Present results of all investigations of possible causes of heterogeneity among study results.	NA
	20d	Present results of all sensitivity analyses conducted to assess the robustness of the synthesized results.	NA
Reporting biases	21	Present assessments of risk of bias due to missing results (arising from reporting biases) for each synthesis assessed.	NA
Certainty of evidence	22	Present assessments of certainty (or confidence) in the body of evidence for each outcome assessed.	NA
DISCUSSION			
Discussion	23a	Provide a general interpretation of the results in the context of other evidence.	Page 13 to 15
	23b	Discuss any limitations of the evidence included in the review.	Page 15
	23c	Discuss any limitations of the review processes used.	Page 15
	23d	Discuss implications of the results for practice, policy and future research.	Page 16
OTHER INFORMATION			
Registration and protocol	24a	Provide registration information for the review, including register name and registration number or state that the review was not registered.	NA
	24b	Indicate where the review protocol can be accessed or state that a protocol was not prepared.	Na
	24c	Describe and explain any amendments to information provided at registration or in the protocol.	NA
Support	25	Describe sources of financial or non-financial support for the review and the role of the funders or sponsors in the review.	Page 16
Competing interests	26	Declare any competing interests of review authors.	Page 16
Availability of data, code and other materials	27	Report which of the following are publicly available and where they can be found: template data collection forms; data extracted from included studies; data used for all analyses; analytic code; and any other materials used in the review.	NA

**Table 2:** Important characteristics of the included studies in the systematic review (*n* = 10).

Study	Year	Country	Study Design	Health Condition	Sample Size	Age (Years)	Gender
(Sobierajska-Rek et al., 2021) [30]	2021	Poland	Survey	Duchenne muscular dystrophy	152	Mean age: 11.00 (SD 7.88)	Boys
(Pamela Frigerio, 2021) [31]	2021	Italy	Survey	Neurodevelopmental Disabilities	128 families	0 to 14	NR
(A. Sobierajska-Rek et al., 2021) [32]	2021	Poland	Online survey	Duchenne muscular dystrophy	69	7.38 years; SD 3.06	boys
(Tanner, Grinde and McCormick, 2021) [33]	2021	Canada	Cross-sectional study	Different conditions	86	8.13 in group 1, 7.21 in group 2 and 6.24 in group 3	Girls: 52 Boys: 34
(Bican et al., 2021) [34]	2021	USA	Retrospective, observational study	Different conditions such as Musculoskeletal impairment, cerebral palsy, movement disorder, delayed milestones, feeding difficulties and prematurity or low birth weight	514	4.3 ± 3.5 years	NR
(Romano, Di Rosa, Tisano, Fabio and Lotan, 2021) [35]	2021	Italy	Case series with multiple evaluation meetings	Rett syndrome	47 goals	17 ± 7.11	Girls
Krasovsky et al., 2021 [36]	2021	Israel	Qualitative survey with Focus groups of experts	All conditions need physical therapy	15	11.31 ± 4.8 years	Both boys and girls
(Provenzi et al., 2021) [37]	2021	Italy	Cross-sectional survey	Autism spectrum disorders, cerebral palsy, neuromuscular diseases, three with rare genetic or malformative syndromes and extremely premature and psychomotor or cognitive delay conditions.	36	5.8 years (range 1–15)	25 boys and 11 girls
Hall et al. [38]	2021	USA	Cross-sectional survey	All conditions need physical therapy in children	259	NA since the respondents were physical therapists	Both
Gagnon et al. [39]	2021	Canada	Single-cohort pilot study	Arthrogryposis multiplex congenital	114 were approached and 10 consented	8 to 21 with a mean age of 16.9 years	Both with equal ratio
(Assenza et al., 2021) [40]	2020	Italy	An observational transversal study	Children with different musculoskeletal and neurodevelopment problems	138	0 to 6 years old for children	NR
(K. Tanner et al., 2020) [41]	2020	USA	Retrospective chart review and survey	Different conditions required physical, occupational and speech therapy	767	NR	NR
(Iannizzotto, Nucita, Fabio, Caprì and Lo Bello, 2020) [42]	2021	Italy	Experiment	Rett genetic syndrome	300	11.31 ± 4.8 years	girls

**Table 3:** Summary of key findings from the included studies in the systematic review (*n* = 10).

Study	Year	Country	Type of Intervention	Key Findings
(Sobierajska-Rek et al., 2021) [30]	2021	Poland	Respiratory physical therapy telerehabilitationVideo with the instructions of respiratory exercises	The average rating for the satisfaction with the therapy was 4.7 out of 5, and it was 4.78 out of 5 for intelligibility. Around 83% of the study participants reported performing the exercises and their caregivers mentioned that it was feasible for their children to perform the recommended exercises a few times a week or daily.
(Pamela Frigerio, 2021) [31]	2021	Italy	Remote therapy	Almost 80.5% of the caregivers showed their satisfaction with telerehabilitation. More than 50% of the families mentioned a higher degree of satisfaction with telerehabilitation.
(A. Sobierajska-Rek et al., 2021) [32]	2021	Poland	The rehabilitation programs	Using physiotherapy can continue home-based rehabilitation. Parents or caregivers accepted instructions or videos given remotely to a greater extent.
(Tanner, Grinde and McCormick, 2021) [33]	2021	Canada	The Canadian Occupational Performance Measure	Canadian Occupational Performance Measure (COPM) is considered a feasible measure as perceived positively by professional pediatric therapists. Around 83% of the therapists agreed or strongly agreed that COPM is easy to use and is reasonable in a given period of time and can be used with children of different conditions.
(Bican et al., 2021) [34]	2021	USA	Physical or occupational therapy with 938 video visits and 150 telephone encounters.	Telerehabilitation services were received by 83.4% of the children, and most occupational and physical therapists (69.1%) agreed that telerehabilitation is as effective as in-person care. Around 93% of the therapists reported that caregivers were available during the sessions and were actively participating.
(Romano, Di Rosa, Tisano, Fabio and Lotan, 2021) [35]	2021	Italy	Rehabilitation program with 47 goals with a customized program for each participant	Out of the total 47 rehabilitative goals set, 78.7% were achieved with around 76.9% of the children’s gross motor function improving with a modest effect (0.604). Parents and caregivers rated the telerehabilitation program satisfactorily with a score of 4.4/5 and general satisfaction was 4.5/5 with adherence to the program being 4.4/5.
Krasovsky et al., 2021 [36]	2021	Israel	Telerehabilitation sessions	Three components including child, parent and sessions were found to explain 71.3% of the variance in the Clinician Evaluation of Telerehabilitation Service. Therapists mentioned that their capability to maintain therapeutic alliance was superior in that they were able to achieve other goals. According to the families, the therapist was highly involved in providing therapy to children regardless of the type of treatment. These results are in the favor of transitioning to telerehabilitation for the pediatric population.
(Provenzi et al., 2021) [37]	2021	Italy	Online Rehabilitation of Children during the Epidemic (EnFORCE) telehealth program that included case-specific tailored telehealth sessions that include parental support and child rehabilitation sessions.	The findings showed that > 80% of the parents mentioned that their children benefited from the program during the lockdown. Parents from 86 to 95% reported increased feelings of engagement, perceived support and self-relevance.
Hall et al. [38]	2021	USA	Telehealth	The study found that a higher degree of engagement and access to telehealth with stable technology is considered a crucial factor for the effectiveness of telehealth. This model of telehealth is supported and correlates to factors such as internet availability and good connection, and the interaction between child and caregiver and resilience of family may play a vital role in moving towards telerehabilitation during the COVID-19 crisis.
Gagnon et al. [39]	2021	Canada	Physiotec (Physiotec Québec Inc.), a software program that can create a home exercise program	The study found that participants performed home exercise program almost twice a week (Mean: 2.04 with 95% CI of 1.25 to 4.08), and they were satisfied with the approach. Out of 15 goals that were set at the beginning of the program, 12 goals were achieved. The study found a statistically significant improvements in pain and comfort for pediatric outcomes (*p*-value: 0.048) on the physical activity questionnaire.
(Assenza et al., 2021) [40]	2020	Italy	Physical, speech, occupational and cognitive-behavioral therapy using telerehabilitation	The findings revealed a correlation among caretakers of children aged 0–3 with feeling overwhelmed with distance care (OR = 3.27), low perception of telerehabilitation for improving objectives (OR = 6.51) and a great perception of feeling supported in establishing regular activity (OR = 2.96). It was concluded that telerehabilitation can be a helpful strategy during a global pandemic.
(K. Tanner et al., 2020) [41]	2020	USA	Telerehabilitation services (speech-language pathology, developmental occupational and physical therapies and sports and orthopedic therapies)	There was a high satisfaction found with 98.97% of the positive responses. Seventy-three point five percent of pre-pandemic patients were returned after implementing telerehabilitation services.
(Iannizzotto, Nucita, Fabio, Caprì and Lo Bello, 2020) [42]	2021	Italy	Eye gaze technology with a videoconferencing software	The preliminary results of the study revealed that videoconferencing software is promising and can be used at a larger base cost effectively without relying on expensive and complicated devices in which children can remotely communicate

## References

[1] Seron P., Oliveros M.-J., Gutierrez-Arias R., Fuentes-Aspe R., Torres-Castro R.C., Merino-Osorio C., Nahuelhual P., Inostroza J., Jalil Y., Solano R. (2021). Effectiveness of Telerehabilitation in Physical Therapy: A Rapid Overview. Phys. Ther..

[2] Rabatin A.E., Lynch M.E., Severson M.C., Brandenburg J.E., Driscoll S.W. (2020). Pediatric telerehabilitation medicine: Making your virtual visits efficient, effective and fun. J. Pediatr. Rehabil. Med..

[3] Damiano D.L. (2006). Activity, Activity, Activity: Rethinking Our Physical Therapy Approach to Cerebral Palsy. Phys. Ther..

[4] Rosenbaum P., Gorter J.W. (2012). The ‘F-words’ in childhood disability: I swear this is how we should think!. Child. Care Health Dev..

[5] Novak I., McIntyre S., Morgan C., Campbell L., Dark L., Morton N., Stumbles E., Wilson S.-A., Goldsmith S. (2013). A systematic review of interventions for children with cerebral palsy: State of the evidence. Dev. Med. Child. Neurol..

[6] Camden C., Wilson B., Kirby A., Sugden D., Missiuna C. (2014). Best practice principles for management of children with developmental coordination disorder (DCD): Results of a scoping review. Child. Care Health Dev..

[7] Zampolini M., Todeschini E., Guitart M.B., Hermens H., Ilsbroukx S., Macellari V., Magni R., Rogante M., Marchese S.S., Vollenbroek M. (2008). Tele-rehabilitation: Present and future. Ann. Dell’istituto Super. Di Sanità.

[8] Cruz V.T., Pais J., Bento V., Mateus C., Colunas M., Alves I., Coutinho P., Rocha N. (2013). A Rehabilitation Tool Designed for Intensive Web-Based Cognitive Training: Description and Usability Study. JMIR Res. Protoc..

[9] Kayyali R., Hesso I., Mahdi A., Hamzat O., Adu A., Gebara S.N. (2017). Telehealth: Misconceptions and experiences of healthcare professionals in England. Int. J. Pharm. Pr..

[10] Edirippulige S., Reyno J., Armfield N., Bambling M., Lloyd O., McNevin E. (2016). Availability, spatial accessibility, utilisation and the role of telehealth for multi-disciplinary paediatric cerebral palsy services in Queensland. J. Telemed. Telecare.

[11] Demers M., Martinie O., Winstein C., Robert M.T. (2020). Active Video Games and Low-Cost Virtual Reality: An Ideal Therapeutic Modality for Children with Physical Disabilities During a Global Pandemic. Front. Neurol..

[12] Iacono T., Stagg K., Pearce N., Chambers A.H. (2016). A scoping review of Australian allied health research in ehealth. BMC Heal. Serv. Res..

[13] Ekeland A.G., Bowes A., Flottorp S. (2010). Effectiveness of telemedicine: A systematic review of reviews. Int. J. Med. Informatics.

[14] Seidman Z., McNamara R., Wootton S., Leung R., Spencer L., Dale M., Dennis S., McKeough Z. (2017). People attending pulmonary rehabilitation demonstrate a substantial engagement with technology and willingness to use telerehabilitation: A survey. J. Physiother..

[15] Russell T.G. (2007). Physical rehabilitation using telemedicine. J. Telemed. Telecare.

[16] Dantas L.O., Barreto R.P.G., Ferreira C.H.J. (2020). Digital physical therapy in the COVID-19 pandemic. Braz. J. Phys. Ther..

[17] International Network of Physiotherapy Regulatory Authorities (2020). Report of the Wcpt/INPTRA digital physical therapy practice task force. World Confed. Phys. Ther..

[18] Camden C., Pratte G., Fallon F., Couture M., Berbari J., Tousignant M. (2019). Diversity of practices in telerehabilitation for children with disabilities and effective intervention characteristics: Results from a systematic review. Disabil. Rehabil..

[19] Bettger J.P., Resnik L.J. (2020). Telerehabilitation in the Age of COVID-19: An Opportunity for Learning Health System Research. Phys. Ther..

[20] Kairy D., Lehoux P., Vincent C., Visintin M. (2009). A systematic review of clinical outcomes, clinical process, healthcare utilization and costs associated with telerehabilitation. Disabil. Rehabil..

[21] Suso-Martí L., La Touche R., Herranz-Gómez A., Angulo-Díaz-Parreño S., Paris-Alemany A., Cuenca-Martínez F. (2021). Effectiveness of Telerehabilitation in Physical Therapist Practice: An Umbrella and Mapping Review with Meta–Meta-Analysis. Phys. Ther..

[22] Dias J.F., Oliveira V.C., Borges P.R.T., Dutra F.C.M.S., Mancini M.C., Kirkwood R.N., Resende R.A., Sampaio R.F. (2020). Effectiveness of exercises by telerehabilitation on pain, physical function and quality of life in people with physical disabilities: A systematic review of randomised controlled trials with GRADE recommendations. Br. J. Sports Med..

[23] Amatya B., Galea M., Kesselring J., Khan F. (2015). Effectiveness of telerehabilitation interventions in persons with multiple sclerosis: A systematic review. Mult. Scler. Relat. Disord..

[24] Johansson T., Wild C. (2011). Telerehabilitation in stroke care—a systematic review. J. Telemed. Telecare.

[25] Prabawa I.M.Y., Silakarma D., Widnyana M. (2021). Telerehabilitation as a physical therapy solution for post-stroke patient in COVID-19 pandemic situations: A review. Multidiscip. J. Sci. Med. Res..

[26] Mark M., Finley J.R., Brazelton A., Kozel C.E., Waterman J., Binkley J. (2021). Implementation and outcomes of telerehabilitation to deliver evidence based physical therapy to breast cancer patients. Rehabil. Oncology..

[27] Page M.J., McKenzie J.E., Bossuyt P.M., Boutron I., Hoffmann T.C., Mulrow C.D., Shamseer L., Tetzlaff J.M., Akl E.A., Brennan S.E. (2021). The PRISMA 2020 statement: An updated guideline for reporting systematic reviews. BMJ.

[28] Brindle M.E., Gawande A. (2020). Managing COVID-19 in Surgical Systems. Ann. Surg..

[29] Potdar R.D., Sahariah S.A., Gandhi M., Kehoe S.H., Brown N., Sane H., Dayama M., Jha S., Lawande A., Coakley P.J. (2014). Improving women’s diet quality preconceptionally and during gestation: Effects on birth weight and prevalence of low birth weight—a randomized controlled efficacy trial in India (Mumbai Maternal Nutrition Project). Am. J. Clin. Nutr..

[30] Sobierajska-Rek A., Mański Ł., Jabłońska-Brudło J., Śledzińska K., Wasilewska E., Szalewska D. (2021). Respiratory Telerehabilitation of Boys and Young Men with Duchenne Muscular Dystrophy in the COVID-19 Pandemic. Int. J. Environ. Res. Public Health.

[31] Pamela Frigerio L.D.M., Sotgiu A., de Giacomo C., Vignoli A. (2021). Parents’ Satisfaction of Tele-Rehabilitation for Children with Neurodevelopmental Disabilities During the Covid-19 Pandemic. BMC Fam. Pract..

[32] Sobierajska-Rek A., Mański Ł., Jabłońska-Brudło J., JŚledzińska K., Ucińska A., Wierzba J. (2021). Establishing a telerehabilitation program for patients with Duchenne muscular dystrophy in the COVID-19 pandemic. Wien. Klin. Wochenschr..

[33] Tanner L.R., Grinde K., McCormick C. (2021). The Canadian Occupational Performance Measure: A Feasible Multidisciplinary Outcome Measure for Pediatric Telerehabilitation. Int. J. Telerehabil..

[34] Bican R., Christensen C., Fallieras K., Sagester G., O’Rourke S., Byars M., Tanner K. (2021). Rapid Implementation of Telerehabilitation for Pediatric Patients During COVID-19. Int. J. Telerehabil..

[35] Romano A., Di Rosa G., Tisano A., Fabio R.A., Lotan M. (2021). Effects of a remotely supervised motor rehabilitation program for individuals with Rett syndrome at home. Disabil. Rehabil..

[36] Krasovsky T., Silberg T., Barak S., Eisenstein E., Erez N., Feldman I., Guttman D., Liber P., Patael S., Sarna H. (2021). Transition to Multidisciplinary Pediatric Telerehabilitation during the COVID-19 Pandemic: Strategy Development and Implementation. Int. J. Environ. Res. Public Health.

[37] Provenzi L., Grumi S., Gardani A., Aramini V., Dargenio E., Naboni C., Vacchini V., Borgatti R. (2021). Engaging with Families through On-line Rehabilitation for Children during the Emergency (EnFORCE) Group Italian parents welcomed a telehealth family-centred rehabilitation programme for children with disability during COVID-19 lockdown. Acta Paediatr..

[38] Hall J.B., Woods M.L., Luechtefeld J.T. (2021). Pediatric Physical Therapy Telehealth and COVID-19: Factors, Facilitators, and Barriers Influencing Effectiveness—a Survey Study. Pediatr. Phys. Ther..

[39] Gagnon M., Merlo G.M., Yap R., Collins J., Elfassy C., Sawatzky B., Marsh J., Hamdy R., Veilleux L.-N., Dahan-Oliel N. (2021). Using Telerehabilitation to Deliver a Home Exercise Program to Youth with Arthrogryposis: Single Cohort Pilot Study. J. Med. Internet Res..

[40] Assenza C., Catania H., Antenore C., Gobbetti T., Gentili P., Paolucci S., Morelli D. (2021). Continuity of Care During COVID-19 Lockdown: A Survey on Stakeholders’ Experience with Telerehabilitation. Front. Neurol..

[41] Tanner K., Bican R., Boster J., Christensen C., Coffman C., Fallieras K., Long R., Mansfield C., O’Rourke S., Pauline L. (2020). Feasibility and Acceptability of Clinical Pediatric Telerehabilitation Services. Int. J. Telerehabil..

[42] Iannizzotto G., Nucita A., Fabio R.A., Caprì T., Bello L.L. (2020). Remote Eye-Tracking for Cognitive Telerehabilitation and Interactive School Tasks in Times of COVID-19. Information.

[43] Mani S., Sharma S., Omar B., Paungmali A., Joseph L. (2017). Validity and reliability of Internet-based physiotherapy assessment for musculoskeletal disorders: A systematic review. J. Telemed. Telecare.

[44] Piga M., Cangemi I., Mathieu A., Cauli A. (2017). Telemedicine for patients with rheumatic diseases: Systematic review and proposal for research agenda. Semin. Arthritis Rheum..

[45] Grona S.L., Bath B., Busch A., Rotter T., Trask C., Harrison E. (2018). Use of videoconferencing for physical therapy in people with musculoskeletal conditions: A systematic review. J. Telemed. Telecare.

[46] DeFre Galea M. (2019). Telemedicine in Rehabilitation. Phys. Med. Rehabil. Clin. N. Am..

[47] Howard I.M., Kaufman M.S. (2018). Telehealth applications for outpatients with neuromuscular or musculoskeletal disorders. Muscle Nerve.

[48] Brennan D., Tindall L., Theodoros D., Brown J., Campbell M., Christiana D., Smith D., Cason J., Lee A. (2010). A Blueprint for Telerehabilitation Guidelines. Int. J. Telerehabil..

